# Real-World Effectiveness of Cladribine for Patients with Multiple Sclerosis: A Sicilian Multicentric Experience (Rewind Study)

**DOI:** 10.2174/1570159X21666230322140711

**Published:** 2024-09-01

**Authors:** Sebastiano Arena, Clara Grazia Chisari, Simona Toscano, Sebastiano Bucello, Luigi Maria Grimaldi, Paolo Ragonese, Sabrina Realmuto, Salvatore Cottone, Davide Maimone, Chiara Finocchiaro, Paola Reitano, Francesco Patti

**Affiliations:** 1 Department “GF Ingrassia” Section of Neurosciences, University of Catania, Catania, Italy;; 2 Multiple Sclerosis Center- PO Muscatello di Augusta, ASP Siracusa, Siracusa, Italy;; 3 Institute Foundation “G. Giglio”, Multiple Sclerosis Centre, Cefalù-Palermo, Italy;; 4 Department of Biomedicine, Neurosciences and Advanced Diagnostics (BiND), University of Palermo, Palermo, Italy;; 5 Multiple Sclerosis Centre, Neurology Unit and Stroke Unit, AOOR “Villa Sofia-Cervello”, United Hospitals, Palermo, Italy;; 6 Azienda Ospedaliera di Rilievo Nazionale e di Alta Specializzazione “Civico Di Cristina e Benfratelli”, Palermo, Italy;; 7 Centro Sclerosi Multipla, UOC Neurologia, ARNAS Garibaldi, Catania, Italy

**Keywords:** Multiple sclerosis, disease modifying therapies, switching therapies, cladribine, moderately active treatment, highly active treatment

## Abstract

**Background:**

Cladribine tablets are a highly effective option for the treatment of relapsing-remitting multiple sclerosis (RRMS).

**Objective:**

The study aims to evaluate the effectiveness of cladribine in a real-world setting.

**Methods:**

This prospective real-world study consecutively screened all RRMS patients from seven different MS centers in Sicily (Italy) who completed the 2-year treatment course of cladribine tablets in the period between 11^th^ March 2019 and 31^st^ October 2021. Data about Expanded Disability Status Scale (EDSS), relapses, previous treatments, adverse events (AEs) and magnetic resonance imaging (MRI) were collected. Patients who were previously treated with other DMTs were further stratified into moderately active treatment (MAT) and highly active treatment (HAT) patients.

**Results:**

A total of 217 patients (70% women, with a mean age of 38.4 ± 11.3 years) were enrolled. Fifty patients (23.0%) were naïve to treatment and 167 (77%) switched from other disease modifying therapies. After the second year of treatment, about 80% were EDSS progression free, 88% remained relapse-free at T24, and 48% of patients were MRI activity-free. Kaplan Meier analyses showed significant differences between MT and HAT in terms of time to first clinical relapse (HR: 2.43, IC 1.02-5.76; *p* = 0.04), time to the first new T1-gadolinium enhancing lesion (HR: 3.43, IC 1.35-8.70; *p* = 0.009) and time to MRI worsening (HR: 2.42, IC 1.15-5.09; *p* = 0.02).

**Conclusion:**

This study confirmed that cladribine is an effective treatment for MS, particularly in naïve patients and those who have switched from MATs.

## 1 INTRODUCTION

Up to now, multiple sclerosis (MS) is an incurable disease and requires long-term treatment with disease modifying therapies (DMTs). Since the Food and Drug Administration (FDA) in the United States and the European Medicines Agency (EMA) in Europe approved the first therapeutic agent, the interferon-beta (IFNβ) [1], more than a dozen of drugs for the treatment of MS have been developed and several new potential molecules will become available soon. Treatment goals in the management of MS have evolved with the approval of increasingly effective novel DMTs, to strongly reduce the relapse rate and achieve the No Evidence of Disease Activity 3 (NEDA-3) status [2, 3]. According to the last approved guidelines, MS treatment should be started as soon as possible, as it is well known that counteracting the inflammatory processes at the earliest in the course of the disease can lead to a lower risk of axonal loss and neurodegeneration and thus of sustained disability [4]. Most available therapies for relapsing-remitting MS (RRMS) typically require chronic intake (daily, weekly or monthly) [5]. However, over the last few years, the MS therapeutic armamentarium has been enriched by the introduction of immune reconstitution therapies (IRTs), which have the potential to induce long-term disease remission with a short-course treatment strategy [6-9]. In addition, significant efforts have been made to improve therapeutic adherence, which is one of the main factors influencing the efficacy of the treatments [10, 11].

Among the most recently approved IRTs, the 2-chloro-2’-deoxyadenosine (2-CdA, cladribine) tablets were recently introduced for the treatment of highly active RRMS. It was first synthetized in 1972, then in 1997, the approval of subcutaneous form was originally rejected, and, finally, in 2017, EMA approved cladribine orally administered for the treatment of active RRMS [12]. Unlike most of the current DMTs, cladribine has multiple mechanisms of action. It acts as an immunomodulator, pushing the cytokine balance toward an anti-inflammatory pattern. Moreover, cladribine has immunosuppressive effects by depleting both the T- and the B-lymphocyte cells [13, 14].

Results from randomized clinical trials and real life studies showed that cladribine tablets significantly reduce relapse rate, the risk of disability progression, and magnetic resonance imaging (MRI) disease activity in MS, particularly in patients with high disease activity [15-21]. In addition, studies on clinically isolated syndromes (CISs) patients documented that cladribine tablets are effective in delaying MS conversion compared with placebo [22].

Thus, these findings have supported the inclusion of cladribine among the highly active treatments (HAT), along with natalizumab (NTZ), fingolimod (FTY), alemtuzumab (ALM) and ocrelizumab (OCR), and thus its possible use also as an exit strategy in case of HAT discontinuation. However, evidence about the effectiveness of cladribine as a treatment choice after second-line HAT is still scarce.

This real-world study aimed to evaluate cladribine effectiveness in a cohort of MS patients from different MS centers in Sicily, Italy.

## 2 MATERIALS AND METHODS

### 2.1 Selection of Patients

We consecutively screened all RRMS patients from seven different MS centers in Sicily (Italy) who were treated with cladribine in the period between 11^th^ March 2019 and 31^st^ October 2021. The study protocol was approved by the Policlinico-Vittorio Emanuele (Catania, Italy) Ethics Committee and by the Ethics Committee of the other participating centers. All patients were informed about the study protocol and signed the informed consent.

Inclusion criteria were the following:

- Age ≥ 18 years old.
- Diagnosis of RRMS according to 2017 McDonald’s criteria [23].
- Capability to understand and sign the specific, informed consent required for the study.

Exclusion criteria were the following:

- Age ≤ 18 years old.
- Diagnosis of Clinically Isolated Syndrome (CIS), Primary or Secondary Progressive Multiple Sclerosis (PPMS or SPMS) according to 2017 McDonald’s criteria [23].
- Patients previously exposed to cladribine in ORACLE-MS, ONWARD and CLARITY trials.

### 2.2 Collection of Data

The following data were collected from the iMed^®^ registry:

- Demographic data: sex, age, weight, height.
- Clinical data: disease duration; symptom(s) at onset; the total number of clinical relapses in the course of the disease, in the previous two years and every six months after exposure to cladribine; any previous use of DMTs; Expanded Disability Status Scale (EDSS) at diagnosis, within three months and every six months from cladribine exposure; adverse events (AEs) occurred after exposure to cladribine with particular attention to Serious AEs (SAEs).
- MRI data: number of encephalic and spinal T2/FLAIR, T1 and T1 Gadolinium enhanced lesion(s) (Gd+) within three months and every six months from cladribine exposure.

Each enrolled patient was anonymized using an alphanumeric code. The extracted data were transferred to electronic databases and analyzed through STATA^®^ 17.0 software [24].

### 2.3 Statistical Analysis

Statistical analysis was performed using STATA^®^ 17.0 software (StataCorp LP, College Station, US) [24]. In descriptive analyses, continuous variables were summarized as mean and standard deviation (SD) or median and interquartile range (IQR), while categorical variables were expressed as percentages. Shapiro-Wilk test was used for the assessment of normal distribution. Student’s t-tests were applied for parametric variables, while nonparametric statistics were used if the distribution of data deviated from normality. The association between two quantitative variables was performed through the Pearson correlation coefficient or Spearman correlation coefficient, depending on the data distribution. A two-sided *p*-value of <0.05 was considered statistically significant.

We compared clinical and demographical data between patients who were naive to treatment before starting cladribine (naïve subgroup) and those who switched from other treatments (switch subgroup). The switch subgroup was further stratified in moderately active treatments (MATs), including interferons, glatiramer acetate, teriflunomide, and dimethyl fumarate) and highly active treatments (HATs), including FTY and NTZ.

The cumulative probability of confirmed EDSS worsening (CEW, defined as ≥ 1 point for EDSS score ≤ 5.0 and as ≥ 0.5 point for EDSS score > 5.0 from baseline that was confirmed) was evaluated. The cumulative probability of CEW was also evaluated in each subgroup (naive *vs*. switch and MAT *vs*. HAT).

Moreover, the cumulative probability of MRI worsening (defined as the presence of ≥ 1 gadolinium-enhanced lesion or the presence of ≥ 1 new or enlarged T2 lesion) was evaluated. The cumulative probability of CEW and MRI worsening was also evaluated in each subgroup (naive *vs*. switch and MAT *vs*. HAT). Not-adjusted and adjusted Annualized Relapse Ratio (ARR) and NEDA-3 were also calculated.

Kaplan-Meier curves were used to estimate the cumulative risk of reaching the following milestones: CEW, first gadolinium-enhanced lesion at last follow-up MRI, MRI worsening at last follow-up, and first clinical relapse in MS patients stratified as naïve or switch at the time of cladribine initiation and as MAT and HAT.

The variables significantly related with time to CEW at 24 months, first gadolinium enhanced lesion at last follow-up MRI, MRI worsening at last follow-up, and first relapse on univariate analysis were considered for inclusion in multivariable analysis. Multivariable Cox proportional hazards models were used to identify demographic and clinical factors significantly and independently associated with the outcomes. The Cox proportional hazard models were corrected for age, sex, mean interval between the suspension of previous DMTs and the cladribine initiation, and disease duration. The null hypothesis was rejected if *p* < 0.05 (also an indicator of statistical significance). The adjusted Hazard Ratios (HRs) and their 95% CI were used to interpret the final model.

All results were compared with those published on CLARITY trial [15] and the following post-hoc analysis [17].

## 3 RESULTS

### 3.1 Patients’ Characteristics

Data from 8,647 patients were obtained from the extraction of all electronic records. Of these, 217 patients (152 women, 70%) met the study's inclusion criteria and were finally enrolled (Tables **1** and **2**).

The mean interval between the two cycles of treatment was 12.9 ± 4.3 months (median 14; range 12-17 months).

A total of 50 patients (23%) were naïve to treatment (naïve group) and 167 (77%) switched from another DMT (switch group). Flow-chart describing patients’ distribution among groups is illustrated in Fig. (**1**). Naïve patients were younger (34.5 ± 11.3 *vs*. 39.6 ± 11.0 years, *p* = 0.004), with shorter disease duration (42.9 ± 34.3 *vs*. 120.9 ± 82.0 months, *p* = <0.0001) compared with switch group. Moreover, naïve patients experienced fewer relapses during the entire course of MS (2.7 ± 1.2 *vs*. 4.5 ± 3.2, *p* = 0.0002) but were more active during the previous two years before exposure to cladribine (2.2 ± 0.9 *vs*. 1.5 ± 1.1, *p*< 0.0001) compared to patients who switched from any DMT. Similarly, EDSS at diagnosis was higher in the naïve group compared to the switch group (2.4 ± 1.4 *vs*. 1.9 ± 1.2, *p* = 0.04). At T0, MRI data showed no differences in the number of brain and spinal T2/FLAIR, T1 and T1-Gd+ lesions between the two groups.

Considering patients switching from previous DMTs, 125 subjects were treated with MATs and 39 with HATs; for 3 patients, it was impossible to trace the previous therapy. Clinical and demographical data were similar among the groups, except for the higher percentage of female patients and the number of relapses in HATs compared with MAT (Tables **3** and **4**).

Among causes of switch, inefficacy was the most important reason (79.0%), followed by patient’s choice (6.6%), presence of any AE (6.0%), Progressive Multifocal Leukoencephalopathy (PML) risk (6.0%), other motivations (2.4%). At the time of analysis, 6 patients (3.22%) interrupted therapy, 2 because of efficacy, 2 for safety, and 2 for other reasons.

### 3.2 Disease Outcomes

**Fig. (2):**
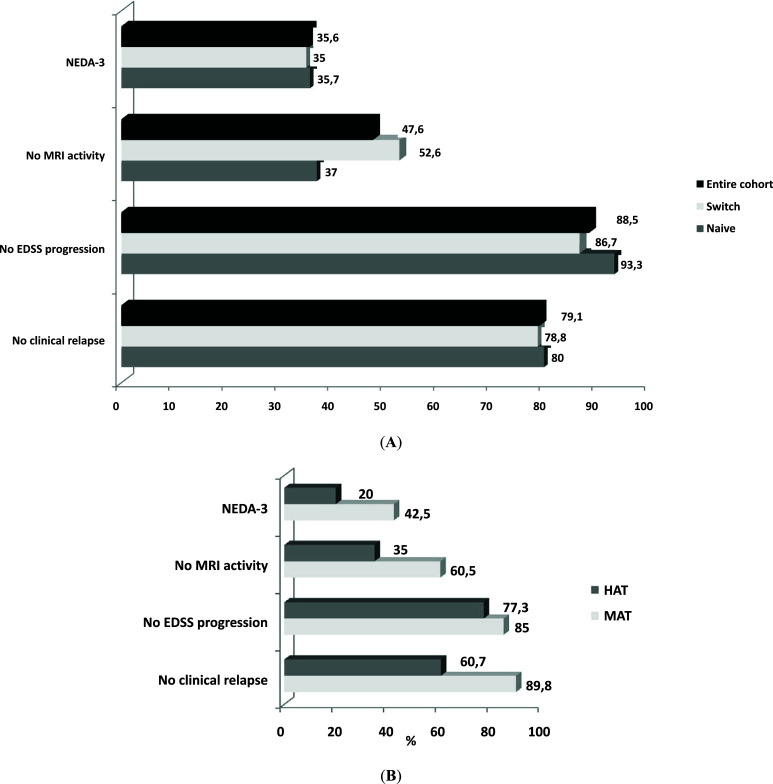
Distribution of patients reaching NEDA-3 status at T12 in naïve and switch groups (**A**) and in MAT and HAT subgroups (**B**). **Abbreviations:** EDSS: Expanded Disability Status Scale, HAT: Highly active treatment, MAT: Moderately Active Treatment, MRI: Magnetic Resonance Imaging, NEDA-3: No Evidence of Disease Activity.

After the first year of treatment with cladribine (T12), about 90% were EDSS progression free, 80% remained relapse-free, and 48% were MRI activity-free (Fig. **2A**). Among 77 (35.5%) patients who reached NEDA-3 could be evaluated, 31 patients (35.2%) reached NEDA-3. Moreover, a higher percentage of NEDA-3 patients was found in the MAT group, even if not statically significant (*p* = 0.08). The percentage of patients relapse-free was higher in the MAT group compared with the HAT (89.8 *vs*. 60.7, *p* = 0.002). No differences emerged in MRI worsening or EDSS free-progression comparison (Fig. **2B**).

**Fig. (3):**
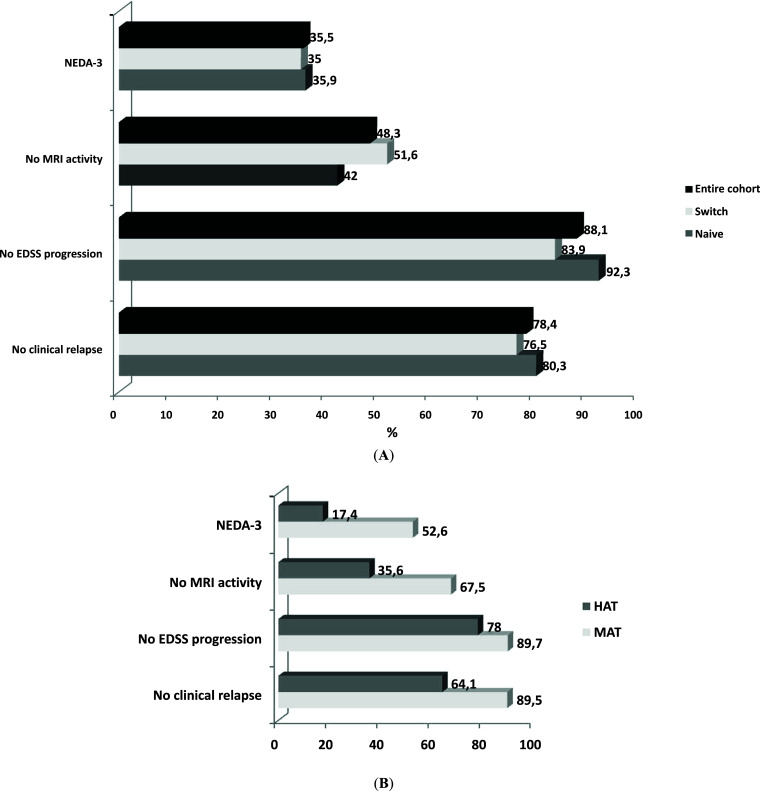
Distribution of patients reaching NEDA-3 status at T24 in naïve and switch groups (**A**) and in MAT and HAT subgroups (**B**). **Abbreviations:** EDSS: Expanded Disability Status Scale, HAT: Highly active treatment, MAT: Moderately Active Treatment, MRI: Magnetic Resonance Imaging, NEDA-3: No Evidence of Disease Activity.

After the second year of treatment (T24), about 80% were EDSS progression free, 88% remained relapse-free at T24, and 48% of patients were MRI activity-free (Fig. **3A**). 77 (35.5%) patients who reached NEDA-3 could be evaluated, 36 patients (39.6%) reached NEDA-3. At T24, a higher percentage of NEDA-3 patients was found in the MAT group (52.6 *vs*. 17.6, *p* = 0.001). Moreover, higher percentages of relapse-free patients and of MRI activity free patients were found in the MAT group compared with the HAT group (67.5 *vs*. 35.6, *p* = 0.003; 89.5 *vs*. 64.1, *p* = 0.001, respectively) (Fig. **3B**).

**Fig. (4):**
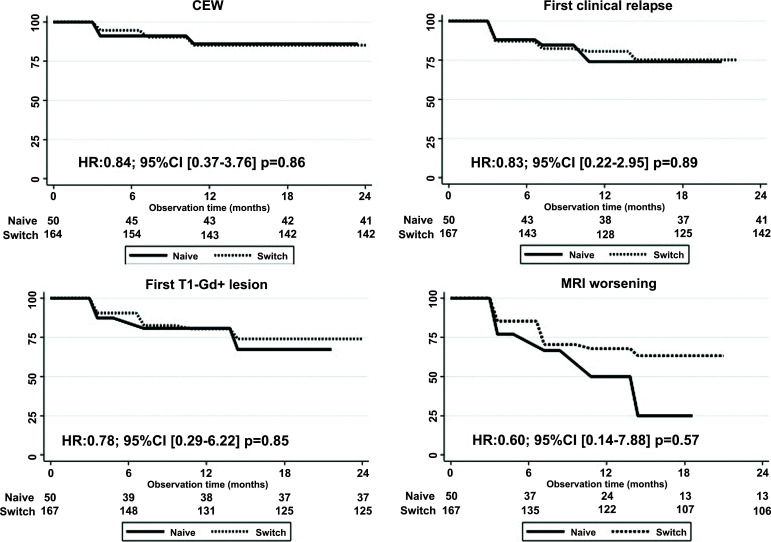
Kaplan-Meier survival curves of disability progression, first clinical relapse and first T1-Gadolinium enhancing lesion(s) in patients naïve to treatment (naïve) and in those who switched from other therapies (swich). **Abbreviations:** CEW: confirmed Expanded Disability Status Scale worsening, Gd: Gadolinium.

Kaplan Meier analyses in naïve and switching patients did not show significant differences in terms of CEW, time to first clinical relapse and time to MRI activity (Fig. **4**).

**Fig. (5):**
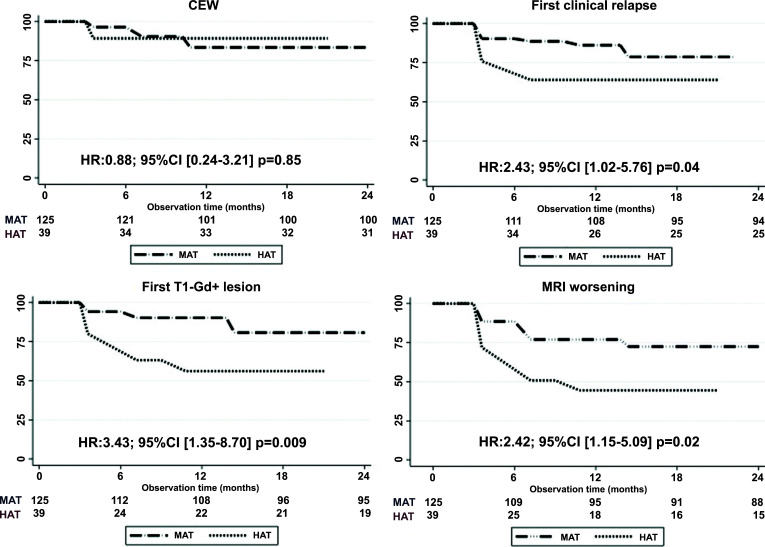
Kaplan-Meier survival curves of disability progression, first clinical relapse and first T1-Gadolinium enhancing lesion(s) in patients who switched from highly active treatments (HAT) and from moderately active treatments (MAT). **Abbreviations:** CEW: confirmed Expanded Disability Status Scale worsening, Gd: Gadolinium, HAT: highly active treatment, MAT: moderately active treatment.

Kaplan Meier analyses in MAT and HAT groups showed significant differences in terms of time to first clinical relapse (HR: 2.43, IC 1.02-5.76; *p* = 0.04), time to the first new T1-gadolinium enhancing lesion (HR: 3.43, IC 1.35-8.70; *p* = 0.009) and time to MRI worsening (HR: 2.42, IC 1.15-5.09; *p* = 0.02). No differences were observed in time to CEW (HR: 0.88, IC 0.24-3.21; *p* = 0.85) (Fig. **5**).

Moreover, the unadjusted ARR at T24 for the entire cohort was 0.18, with a lower value in patients switching from MAT (0.11), compared to those switching from a HAT (0.34). Analysis of adjusted ARR at T24 showed similar results (Table **5**).

### 3.3 Comparison with CLARITY and Post-Hoc Analysis

Among the 433 patients enrolled in the CLARITY study and exposed to cladribine tablets, 113 (26.1%) patients were previously treated with other DMTs. The sub-analysis of this group documented that DMTs were, in most cases, intramuscular IFN-beta-1a (11.2% of patients), subcutaneous IFN-beta-1b (10.6% of patients), subcutaneous IFN-beta-1a (9.4% of patients), and subcutaneous GA (6.5% of patients) [15-17]. Patients who were included in the CLARITY trial showed lower percentages of patients who were previously treated with other DMTs and of patients with high disease activity compared to our cohort. Moreover, ARR values were similar between the two cohorts, except for the HAT subgroup, in which ARR was higher in our study compared to CLARITY. Comparison of demographic and clinical characteristics between CLARITY and REWIND cohorts was illustrated in Table **6**.

### 3.4 Adverse Events

A total of 24 AEs were observed, 17 (70.8%) in the switch and 7 (41.2%) in the naïve group. Infections were the most frequent (14, 58.3%), followed by thyroid function testing abnormalities (3, 12.5%), dermatological complications (2, 8.3%), increased liver enzymes, hyperuricemia and depression (1 case each, 4.2%). Data about the incidence of lymphopenia at each time point were illustrated in Fig. (**6**).

In addition, three serious AEs potentially associated with cladribine treatment were reported, all in the switch group:

1) A “moderately differentiated keratinizing ulcerated squamous cell carcinoma” (pathological stage pT1A) in a 54 years old male with EDSS 4.5 at T6 required local surgery. The patients also performed a positron emission tomography, which was negative.
2) Systemic infection in a 56 years old woman with EDSS 6.0 at T12. Lymphocytes counts at the event were 800/mmc. During hospitalization, the patient was treated with antibiotics, antivirals and recombinant human factor stimulating granulocyte and macrophage colonies and recovered completely.
A pancreatic failure, of unidentified cause, in a 51 years old female with EDSS 3.5 was diagnosed at T12 and prompted the precautionary interruption of cladribine at year 2.

3) Five cases of severe acute respiratory syndrome coronavirus 2 (SARS-CoV2) infections were present among AEs (20.8%). No patient required hospitalization, respiratory support or domiciliary oxygen therapy.

## 4 DISCUSSION

This real-world prospective study confirmed that cladribine is an effective treatment for MS, particularly in naïve patients and those who have switched from MATs. About 90% of patients who have completed the second year of treatment were EDSS-progression free and 80% experienced no relapse.

Our results are aligned with the data that emerged from the pivotal and real-life studies demonstrating the high efficacy profile of cladribine tablets for treating patients with relapsing MS [2, 18-20, 22-25]. Preliminary results from 8-11 years of follow-up of the CLASSIC-MS study also highlighted the long-term efficacy in patients who received cladribine in the ORACLE study. Authors claimed that patients treated with cladribine experienced sustained long-term mobility with delayed conversion to MS and reduced frequency of confirmed second relapse compared to those who were never exposed to cladribine [26]. Similarly, a recent MSBase study (GLIMPSE) comparing the effectiveness of cladribine compared to other oral DMTs showed that relapse and discontinuation outcomes significantly favoured cladribine over other oral DMTs [27].

On the other hand, we found a low percentage (about 48%) of patients who were MRI activity free at 12 and 24 months. Indeed, previous studies have shown reductions in T1 Gd+ lesion counts of between 85% and 92% over 2-year study periods in patients treated with cladribine tablets [28-31]. However, it should be noted that MRI results from these studies were obtained through the analysis of MRI scans performed during the CLARITY or MAGNIFY-MS trials, thus, including thorough assessments and standardized acquisition methods. In contrast, our real-world study included MRI data obtained from different MRI machines and evaluated by neurologists from several Sicilian MS centers. This may influence our results and potentially underestimate the MRI findings.

In addition, our survival analysis documented no differences between naïve and switching patients in terms of EDSS progression, clinical relapse and MRI worsening. However, among patients who have switched from other DMTs, the MAT group showed better clinical outcomes compared to HAT. These results were also confirmed by the higher percentage of patients reaching NEDA-3 after the first year of therapy in the MAT group, with a higher percentage of patients free from disease progression, clinical relapses and radiological worsening. Similarly, ARR analysis showed the lowest values in MAT patients compared to naïve and HAT patients.

Our findings are consistent with the post-hoc analysis of the CLARITY extension study showing that patients treated early with cladribine exhibited a significantly higher prevalence of disability improvement at year 2, and thus, confirming that the earlier use of highly active therapies, such as cladribine, is associated to more favorable clinical outcomes [32].

According to our results, cladribine expresses the maximum of its effectiveness when used as the first treatment choice in naïve patients or after first-line therapies failure, while the question still open is if cladribine may represent a potential exit strategy therapy from treatment with NTZ or FTY. Indeed, although cladribine is included among the HATs, clinicians are inclined to consider it less efficacious than other second line DMTs such as OCR, NTZ, ALM and ofatumumab [33]. In this respect, data from the current literature are often conflicting. Some authors underlined the risk of disease flare-ups after NTZ discontinuation, which is poorly reduced by the use of powerful drugs [34]. Similarly, several case reports have documented the risk of disease recurrence in patients who switched to cladribine from FTY [35, 36], probably driven by the autoreactive cells sequestrated within lymph nodes that evade the cladribine effects on top of a delay of re-circulating regulatory T cells [37, 38]. On the contrary, a case series of 17 patients suggested that cladribine may represent a good exit strategy in patients who have to discontinue NTZ due to PML risk [39].

The pivotal CLARITY trial and the following post-hoc analysis led to the thorough characterization of the safety and efficacy profile of cladribine tablets in MS and contributed to the final approval by EMA and FDA [40, 41]. Compared to our data, CLARITY included a lower percentage of patients (26.1%) who switched from other DMTs, with 39 patients previously exposed to highly active drugs (FTY, NTZ) [42]. On this basis, it is conceivable that patients who were included in the CLARITY cohort had a relatively lower disease activity compared to those participating in REWIND; this discrepancy is reflected by the higher ARR values observed in the subgroup of patients who switched from HAT in our study compared to CLARITY. Notably, patients included in the pivotal studies of other approved HATs, such as FTY and NTZ, presented a higher disease activity, as demonstrated by higher ARRs showed in these studies [25, 43].

The safety profile for cladribine tablets has been well-characterized in a wide population of RRMS patients. According to several studies, no increased risk for infections, except for a higher incidence of herpes zoster, was reported. More importantly, no increase in malignancy rates for cladribine tablets relative to placebo was demonstrated [12, 20, 44-47]. Similar to a recent real-world study of an Italian cohort, grade III and IV lymphopenia incidence was low and similar to those observed in clinical trials [44, 48].

Moreover, all three observed SAEs (of which at least two were likely related to the treatment) were reported in the switch group. In particular, all patients who presented a SAE were over 50 years old with a moderate level of disability (the minimum EDSS was 3.5). These data may suggest that switching to cladribine in the presence of advanced age or high disability level may be more frequently associated with AEs, in some cases even severe. In line with our data, a recent paper showed a higher frequency of AE and infections during cladribine treatment among aged and disabled people with MS.

This study has several limitations. First, the retrospective design and the limited sample of patients included may have reduced the statistical power of our results. Second, the lack of data on MRI exams in some patients may have reduced the percentage of patients reaching NEDA-3 in our study. Moreover, as brain volume and the number of white matter lesions could influence long-term disability outcomes, longitudinal studies, including the largest samples and prospective MRI evaluations, are needed to draw some conclusions about the real-life efficacy of cladribine tablets in the MS population.

## CONCLUSION

REWIND study analysed one of the largest cohorts of MS patients treated with cladribine tablets in a real-world setting. Our results suggest that cladribine appears to be an effective treatment option, particularly in patients naïve to treatment and in those previously treated with MAT. In light of our results, longitudinal studies on larger cohorts are needed in order to identify those factors that may predict cladribine response in patients who switched from HATs. Moreover, results from our study may drive clinicians to use cladribine earlier in MS treatment management compared with other drugs, also in light of the higher risk of AEs in aged and high-disabled patients. In conclusion, cladribine tablets are a good therapeutic option during the early phase of the disease, thanks to the better therapeutic burden (two weekly treatment courses one year apart) and the potential to keep the patient treatment-free for several years.

## Figures and Tables

**Fig. (1):**
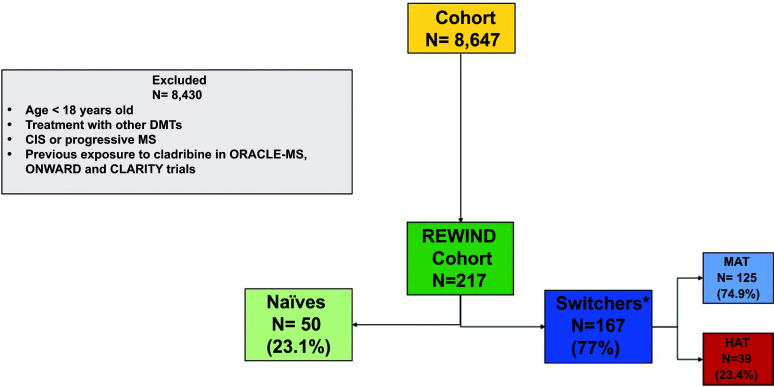
Flow-chart of patients’ distribution among groups. Naïve: patients naïve to treatment before starting cladribine treatment; switch: patients who switched from other disease modifying therapies; HAT: patients previously treated with highly active treatment, MAT: patients previously treated with moderately active treatment. **Note:** *for 3 patients, data about previous therapies were unavailable.

**Fig. (6):**
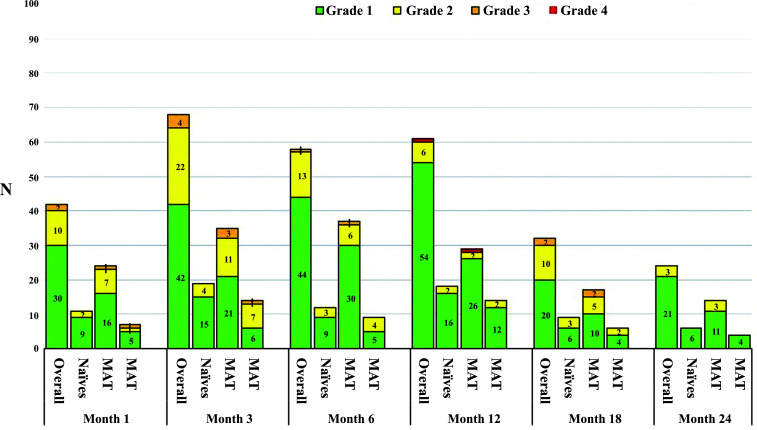
Grade distribution of lymphopenia according to the CTCAE score. Common Terminology Criteria for Adverse Events (CTCAE) score for lymphopenia: grade 1 = < 800/mm^3^; grade 2 = < 800-500/mm^3^; grade 3 = < 500-200/mm^3^; grade 4 = < 200/mm^3^. **Abbreviations:** HAT: highly active treatment, MAT: moderately active treatment.

**Table 1:** Demographical and clinical characteristics of the study cohort.

-	**Total** **(n = 217)**	**Naïve** **(n = 50)**	**Switch** **(n = 167)**	***p* Value**
Age (y); mean ± SD	38.4 ± 11.3	34.5 ± 11.3	39.6 ± 11.0	0.004
Females; n (%)	152 (70.0)	35 (70.0)	117 (70.0)	1.0
Weight (Kg); mean ± SD	68.2 ± 15.7	68.5 ± 14.8	68.1 ± 15.9	0.9
Height (cm); mean ± SD	168.2 ± 9.7	167.8 ± 9.3	168.6 ± 10.4	0.9
Disease duration (months); mean ± SD	102.7 ± 80.7	42.9 ± 34.3	120.9 ± 82.0	<0.0001
Total n. of relapses; mean ± SD	4.1 ± 3.0	2.7 ± 1.2	4.5 ± 3.2	0.0002
Annualized relapse rate 2 years before CLD initiation; median (range)	0.55 (0.16-0.67)	0.51 (0.16-0.58)	0.59 (0.19-0.67)	0.001
EDSS at diagnosis; mean ± SD	2.1 ± 1.3	2.4 ± 1.4	1.9 ± 1.2	0.04
EDSS T0*; mean ± SD	2.6 ± 1.7	2.2 ± 1.3	2.8 ± 1.7	0.05
EDSS T6; mean ± SD	2.3 ± 1.6	1.9 ± 1.2	2.5 ± 1.7	0.05
EDSS T12; mean ± SD	2.3 ± 1.7	1.8 ± 1.3	2.5 ± 1.8	0.06
EDSS T18; mean ± SD	2.5 ± 1.9	2.2 ± 1.7	2.7 ± 2.0	0.6
EDSS T24; mean ± SD	2.1 ± 1.6	1.6 ± 1.5	2.2 ± 1.6	0.3

**Note:** *T0 corresponds to the maximum time of 3 months prior to assuming the first cycle of cladribine.

**Abbreviations:** EDSS: Expanded Disability Status Scale, SD: standard deviation.

**Table 2:** Radiological characteristics of the study cohort^a^.

-	**Total ** ** (n = 217)**	**Naïve** ** (n = 50)**	**Switch** ** (n = 167)**	***P* Value**
MRI at T0*	-	-	-	-
Brain T2 lesions	34.4 ± 27.8	36.6 ± 27.2	33.5 ± 28.1	0.5
Brain T1 lesions	11.5 ± 12.9	12.0 ± 13.1	11.3 ± 12.9	0.8
Brain Gd+ lesions	1.0 ± 1.5	0.8 ± 1.6	1.0 ± 1.5	0.8
Spinal T2 lesions	2.7 ± 2.0	2.9 ± 2.2	2.7 ± 1.9	0.8
Spinal Gd+ lesions	0.3 ± 0.6	0.4 ± 0.7	0.2 ± 0.6	0.8
MRI at T12	-	-	-	-
Brain T2 lesions	38.8 ± 28	34.9 ± 26.1	40.8 ± 29.2	0.2
Brain T1 lesions	15.1 ± 11.8	14.5 ± 7.6	16.2 ± 13.3	0.4
Brain Gd+ lesions	0.6 ± 2.6	0.2 ± 0.5	0.8 ± 3.1	0.2
Spinal T2 lesions	2.9 ± 2.0	2.7 ± 1.5	3.1 ± 2.3	0.2
Spinal Gd+ lesions	0.03 ± 0.2	0.02 ± 0.3	0.04 ± 0.2	0.7
MRI at T24	-	-	-	-
Brain T2 lesions	37.7 ± 29.6	34.2 ± 15.9	41.4 ± 35.0	0.2
Brain T1 lesions	16.6 ± 13.9	14.0 ± 6.7	18.0 ± 16.2	0.09
Brain Gd+ lesions	0.2 ± 0.4	0.3 ± 0.6	0.4 ± 0.5	0.08
Spinal T2 lesions	3.2 ± 2.0	3.5 ± 1.3	3.0 ± 2.4	0.2
Spinal Gd+ lesions	0.09 ± 0.3	0.3 ± 0.5	0.0	0.03

**Note:** ^a^data are expressed as mean±standard deviation.

*T0 refers to 3 months prior to assuming the first cycle of cladribine.

**Abbreviations:** Gd+: Gadolinium enhanced lesions, MRI: magnetic resonance imaging.

**Table 3:** Demographical and clinical data of patients who switched from other therapies.

-	**MAT** ** n = 125**	**HAT** ** n = 39**	***P* value**
Age (years); mean ± SD	40.0 ± 11.7	38.1 ± 9.1	0.4
Females; n (%)	81(64.8)	33(84.6)	0.02
Weight (Kg); mean ± SD	68.0 ± 16.5	65.8 ± 13.0	0.4
Height (cm); mean ± SD	168.2 ± 10.0	169 ± 10.8	0.8
Disease duration (months); mean ± SD	114.6 ± 80.9	134.4 ± 76.6	0.2
Total n. of relapses; mean ± SD	4.1 ± 2.3	5.9 ± 5.2	0.003
Annualized relapse rate 2 years before CLD initiation; median (range)	0.62 (0.22-0.87)	0.56 (0.29-0.86)	0.2
EDSS at diagnosis; mean ± SD	1.9 ± 1.1	2.2 ± 1.5	0.4
EDSS T0*; mean ± SD	2.7 ± 1.7	2.8 ± 1.9	0.8
EDSS T6; mean ± SD	2.4 ± 1.7	2.5 ± 1.8	0.8
EDSS T12; mean ± SD	2.4 ± 1.8	2.6 ± 1.8	0.5
EDSS T24; mean ± SD	2.3 ± 1.5	2.1 ± 1.9	0.5

**Note:** *T0: within 3 months from starting cladribine treatment.

**Abbreviations:** EDSS: Expanded Disability Status Scale, Gd: Gadolinium, HAT: highly active treatments; MAT: moderately active treatments; MRI: magnetic resonance imaging.

**Table 4:** Radiological characteristics of patients who switched from other therapies^a^.

-	** MAT** ** n = 125**	** HAT** ** n = 39**	***P***
MRI at T0*	-	-	** -**
Brain T2 lesions	33.1 ± 24.7	35.9 ± 35.6	0.6
Brain T1 lesions	10.4 ± 8.9	13.6 ± 19.5	0.2
Brain Gd+ lesions	1.1 ± 1.5	1.0 ± 1.4	0.7
Spinal T2 lesions	2.8 ± 2.0	2.5 ± 1.8	0.4
Spinal Gd+ lesions	0.3 ± 0.6	0.2 ± 0.5	0.3
MRI at T12	-	-	-
Brain T2 lesions	38.4 ± 25.1	45.6 ± 36.5	0.2
Brain T1 lesions	15.2 ± 10.6	18.4 ± 18.1	0.2
Brain Gd+ lesions	0.3 ± 1.4	1.9 ± 5.1	0.09
Spinal T2 lesions	3.2 ± 2.3	2.8 ± 2.0	0.3
Spinal Gd+ lesions	0.02 ± 0.2	0.1 ± 0.3	0.03
MRI at T24	-	-	-
Brain T2 lesions	42.5 ± 39.9	38.0 ± 24.0	0.5
Brain T1 lesions	24.2 ± 17.4	26.0 ± 13.9	0.6
Brain Gd+ lesions	0.4 ± 0.5	0.3 ± 0.6	0.3
Spinal T2 lesions	3.0 ± 2.6	3.0± 2.1	0.9
Spinal Gd+ lesions	0	0	NA

** Note:** ^a^Data are expressed as mean ± standard deviation (SD).

*T0: within 3 months from starting cladribine treatment.

** Abbreviations:** Gd: Gadolinium, MRI: magnetic resonance imaging, NA: not applicable.

**Table 5:** Unadjusted and adjusted Annualized Relapse Rate (ARR) at T24.

-	**Total Cohort**	**Naive**	**Switch**	**MAT**	**HAT**
Total N of relapses	38	12	26	13	13
Patient-days	79,500	19,741	59,759	44,817	14,143
Unadjusted ARR	0.17	0.22	0.16	0.11*	0.34
Adjusted ARR median, (range)	0.22 (0.12-0.32)	0.21 (0.07-0.34)	0.22 (0.10-0.35)	0.12* (0.03-0.22)	0.60 (0.14-1.07)

**Note:** *p value <0.05 *versus* HAT.

**Abbreviations:** ARR: Annualized relapse rate, HAT: highly active treatment, MAT: moderately active treatment.

**Table 6:** Comparison between REWIND and CLARITY cohorts.

**-**		**REWIND** **(n = 217)**	**CLARITY Placebo** **(n = 437)**	**CLARITY ** **3.5 mg/Kg (n = 433)**	***p* Value**
Age (years); mean ± SD		38.4 ± 11.3	38.7 ± 9.9	37.9 ± 10.3	0.6
Female N (%)		152(70.0)	288(65.9)	298(68.8)	0.3
Weight (Kg); mean ± SD		68.2 ±15.7	70.3 ± 15.4	68.1 ± 14.6	0.5
Disease duration (months); mean ± SD		102.7 ± 80.7	108.0 ± 90.0	96.0 ± 86.4	0.1
Brain Gd+ lesions at T0*; mean ± SD		1.0 ± 1.5	0.8± 2.1	1.0 ± 2.7	0.9
EDSS at T0; mean ±SD		2.6 ± 1.7	2.9 ± 1.3	2.8 ± 1.2	0.9
N. of switchers **N (%)		167(67.0)	142(32.5)	113(26.1)	0.01
Patients with high disease activityN (%)		182(83.9)	149(34.1)	140(32.3)	0.001
Adjusted Annualized Relapse Rate at 2 years; median (range)	Entire Cohort	0.22 (0.12-0.32)	0.33 (0.29-0.38)	0.14 (0.12-0.17)	0.3
	Naive	0.21 (0.07-0.34)			
	Switchers**	0.22 (0.10-0.35)			
	*MAT*	0.12 (0.03-0.22)			
	*HAT*	0.60 (0.14-1.07)	0.47 (0.40-0.57)°	0.33 (0.23-0.48)°	0.03

Note: *patients who experienced at least one clinical relapse in the previous year and with at least 1 T1 Gd+ lesion during treatment with other disease modifying therapies; patients naïve to treatment, who experienced 2 or more clinical relapses in the previous year.

**patients who switched to cladribine from other disease modifying therapies.

**°**results from a post-hoc analysis.

**Abbreviations:** EDSS: Expanded Disability Status Scale, Gd+: gadolinium-enhancing lesion, HAT: highly active treatment, MAT: moderately active treatment.

## Data Availability

The data used in this study will be available from the corresponding author [FP], upon reasonable request.

## LIST OF ABBREVIATIONS

ALM: Alemtuzumab
ARR: Annualized Relapse Ratio
CISs: Clinically Isolated Syndromes
DMTs: Modifying Therapies
FTY: Fingolimod
HATs: Highly Active Treatments
IRTs: Immune Reconstitution Therapies
MATs: Moderately Active Treatments
MRI: Magnetic Resonance Imaging
MS: Multiple Sclerosis
OCR: Ocrelizumab
